# The impact of COVID-19 on kidney transplant care

**DOI:** 10.3389/fmed.2022.1093126

**Published:** 2023-01-09

**Authors:** Chi Zhang, Lavanya Kodali, Girish Mour, Caroline Jadlowiec, Amit K. Mathur

**Affiliations:** ^1^Division of Transplant Surgery, Mayo Clinic Arizona, Phoenix, AZ, United States; ^2^Robert D. and Patricia E. Kern Center for the Science of Health Care Delivery Mayo Clinic Rochester, Rochester, MN, United States; ^3^Division of Nephrology, Mayo Clinic Arizona, Phoenix, AZ, United States

**Keywords:** kidney transplant, solid organ transplant, care delivery, COVID-19, telemedicine, healthcare access

## Abstract

The SARS-CoV-2 virus precipitated the coronavirus 2019 (COVID-19) pandemic, which placed considerable strain on healthcare systems and necessitated immediate and rapid alterations in the delivery of healthcare. In the transplant population, COVID-19 directly impacts an inherently vulnerable population in the setting of immunosuppression and co-morbidities, but also further complicates the clinical evaluation and management of kidney transplant candidates and recipients in a strained healthcare environment being challenged by the pandemic. Many transplant centers around the world saw mortality rate spikes in organ recipients related to COVID-19, and changes in care delivery abound. This review evaluates the care of the kidney transplant patient through all phases of the process including pre-operative evaluations, perioperative care, post-transplantation considerations, and how the global pandemic has changed the way we care for our patients.

## Access to kidney transplantation: Current trends and gaps in care

Despite nearly 100,000 patients on the US waiting list, kidney transplant (KT) remains the most optimal therapy for end-stage renal disease (ESRD), with survival, quality of life, and cost advantages over other forms of renal replacement therapy (RRT) (1, 2). Though KT has clear benefits over other RRT modalities, the rate of pre-emptive KT was only 3% in the United States in 2016 (3, 4). A critical barrier in access to transplant is a lack of timely referral to the transplant center (5). Multiple demographic and system-level characteristics, many of which are non-modifiable, have been associated with lower rates of KT referral, including older candidate age, minority race/ethnicity, lower socioeconomic status, employment status, medical literacy, and dialysis unit ownership (6–12).

Further expansion of the KT waitlist candidate pool is critical in providing equitable care to the ESRD community. While the US has the most successful deceased donation program in the entire world, there is tremendous variability in organ donation rates, kidneys recovered, and recovered kidneys utilized for transplant across the US (13). Non-utilization of kidneys recovered from deceased donors is a critical issue that varies based on organ procurement organization (OPO) performance, transplant center acceptance patterns, and the intrinsic health status of donors at the time of death (14). Greater emphasis on living donor kidney transplantation is a potential solution to these shortcomings in deceased donor system and has the potential to address the needs of the entire waitlist. Living donation had increased in the US in the years leading up to the pandemic, culminating in 6,866 living donor transplants in 2019, but only accounted for 38% of all US KTs (13). Underlying socioeconomic status may limit current donor availability and recipient access. Within the same geographic area, African American living donor candidates were less likely to progress from referral to donation and some have shown that African American donor candidates have a longer median time from screening to donation compared to other races (15). Despite near universal access to Medicare coverage for KT, African American patients and those from a lower socioeconomic class are less likely to undergo LDKT and have a lower likelihood of being listed for transplant (16–21).

The COVID-19 pandemic exacerbated an already stressed system and highlight deficiencies in all phases of care, resulting in innovation. This review focuses on the impact of COVID-19 on kidney donation and transplantation.

## Pre-transplant care

### Setting the stage

Pre-transplant care entails a series of interwoven processes that begins with evaluation at a transplant center, involves a myriad of diagnostic tests and clinical evaluations, and the provision of dialysis care during this time when the patient is away from their normal home institutions. The subsequent decision on whether to list the patient for transplant involves distillation of medical, surgical, and psychosocial evaluations and the input of several professionals from a multidisciplinary transplant team. The time from referral for transplant to evaluation initiation is highly variable across centers, and the entire process can extend to more than a year (22). Waitlisted candidates must wait, either to receive a kidney from their living donor or for a potential deceased donor kidney. This process can often be protracted. In the absence of a living donor, patients in the United States can wait over 7 years for a deceased donor KT, depending on their donor-specific antibody profile (23, 24).

With the onset of the pandemic, pre-transplant care delivery underwent a substantial disruption of normal processes. Due to the desire to have patients avoid excessive interactions with the hospital, care needed to be streamlined to achieve the same diagnostic accuracy to judge fitness for transplant but keep patients safe from the potential spread of the virus.

### Organ donation: Living donor and deceased donor processes

Living kidney donor evaluation is detailed and known for its rigor, which is aimed at minimizing donor risk. While there is some variability in transplant center approaches, living donor evaluations begin with screening, initial interviews with independent living donor advocates, social workers, and sometimes clinical team members. Blood and tissue typing are also essential components. If deemed suitable to proceed with an evaluation, the potential donor would proceed to subsequent testing which would include medical and surgical consultation, and diagnostic batteries that include blood pressure monitoring, blood and urine tests, cardiac function screening (EKG and echocardiogram), cross-sectional imaging, and other consultations with specialists in social services, nutrition, nursing, urology, transplant psychology and psychiatry, and pharmacy. The multidisciplinary team will approve them to move forward with donation or rule them out due to a variety of reasons, but donors may withdraw from the process at any time including right up to the time of surgery. These evaluations occur in parallel or in series with recipient evaluations. Once a living donor is approved, donors may donate directly to their intended recipient if one is designated and approved, or they may be entered into a paired kidney exchange to optimize donor-recipient matching (25). While the extensive evaluation process is rooted in safety for both the donors and for the recipient, the process can be perceived as arduous.

When there is not a living donor available, most others receive allografts from deceased donors. Brain dead donors present to the hospital antemortem and are declared braindead after a series of provocative bedside neurologic tests to suggest irreversible cessation of brainstem activity. Unlike brain death donors, donation after cardiac death donors often suffer severe injury but do not fulfill the criteria for brain death. However, if by family decision or by advance directive, they may still be able to donate once the family decides to withdraw life support. In both scenarios, the patients are referred to organ procurement organizations (OPOs) and organ donation authorization is pursued. Donor histories, diagnostic laboratory tests (including biopsy), and cross-sectional imaging are obtained, and families are counseled. Once consent is obtained and the patients are deemed medically appropriate for donation, organs are subsequently offered to transplant programs. If accepted, the procurement team arrives to the operating room and begins recovery (26). COVID-19 presented a disruption to the process as donors required testing prior to organ allocation. A positive test, depending on the transplant center, the availability of a willing recipient, and the timing within the pandemic, could obfuscate the donation process entirely. However, the universal acknowledgment of COVID-19 and the requirement to test all deceased donors in the US within the first months of the pandemic was critically important in allowing for the continuation of deceased donations as regulations on procurement loosened with time and with the greater availability of data on COVID positive donors (27).

Challenges in living donor and deceased organ donation were certainly exacerbated at the advent of the COVID-19 pandemic. Transplant centers around the world saw a decline in both deceased and living kidney donation by up to 90% in some European countries (28–34). For living donors, there was concern for contracting COVID-19 in the process of an elective procedure. Hospitals were being overrun by symptomatic COVID-19 patients requiring general and intensive care, which disincentivized time-intensive deceased donor processes including donation authorization, clinical management, and placement with transplant centers. The COVID-19 pandemic ushered in a novel and unseemly dilemma. Clinical teams felt they were choosing between two evils: Accepting waitlist mortality by post-poning transplantation or accepting potential detrimental outcomes related to post-operative recovery and immunosuppression from the virus itself or delays in care (35). Clearly, process changes were necessary and telemedicine helped bridge barriers (36).

### The rapid adoption of telemedicine

As the pandemic evolved in the early months of 2020, it was evident that all health care including transplant and living donor care needed to evolve. The high potential of droplet transmission of the SARS-CoV-2 virus between individuals represented a threat to both patients and to providers. In order to ensure the continuation of care consultations, hospitals and clinics around the globe developed telemedicine protocols using multiple platforms (37, 38). Telemedicine included a variety of types of patient-provider interactions including telephone consultations/visits, virtual visits using video conferencing, and other schemes. The demand for remote and virtual visits with physicians, which was relatively boutique prior to the pandemic, rapidly became a daily practice (39–41).

In transplantation, telemedicine adoption filled a tremendous gap at a time of uncertainty and global paranoia. The outcomes of COVID-19 disease were still evolving, and geographic hot-spots of disease came and went, all while overwhelming healthcare facilities and providers. There was tremendous worry about the impact of COVID-19 disease on immunocompromised individuals that emerged from the very first reports of the virus. Telemedicine helped address the continued need to provide care in all phases of transplant care as needed while minimizing the risk of viral transmission to pre-transplant, peri-transplant, and post-transplant patients, as well as health care providers (42–48).

### Evaluation of living donor and transplant candidates with the implementation of telemedicine

Transplant centers started the living donor evaluation process remotely, especially in patients who had limited capacity to travel. Meetings with living donor advocates, preoperative education, and informed consent could be conducted virtually, while laboratory and imaging tests be completed locally or at the transplant center using social distancing, masking, and droplet precautions. Transplant centers re-evaluated their protocols with an eye toward streamlining the entire living donor candidate care paradigm. Consultations were consolidated as multiple specialists could be seen concurrently. With the evaluation complete, only if a patient is deemed to be a suitable donor by the transplant team would the donor candidate be required to travel to the transplant center a few days prior to the scheduled kidney transplant surgery. Telemedicine and virtual donor evaluation demonstrated multiple potential benefits. For living donor candidates, virtual evaluation was financially advantageous as it required minimal travel in the evaluation phase. For the transplant center, telemedicine could also increase access to LDKT because more evaluations can be completed (36).

A similar process applied to transplant candidate evaluation. Preoperative laboratory tests could be conducted locally and shared digitally with the transplant center through the electronic medical record and diagnostic lab system inputs. Transplant evaluations were completed with one or sometimes no in-person visits, depending on the clinical history and risk. Some centers would list patients inactive on the waiting list once the evaluation was complete, and would activate them after an in-person visit and clinical exam (49).

### Use of video conferencing technologies within transplant hospitals

Healthcare lags in the adoption of new technologies compared to other industries. Prior to the pandemic, large corporations and businesses conducted their work frequently using digital video-based platforms. These were not common technologies used in the daily work of transplant clinicians. However, since the advent of clinical transplantation, multidisciplinary transplant teams have met together physically to discuss the selection and listing of transplant candidates and approval of living donors. It is a near-universal activity in transplant centers. Administrative processes often required in-person meetings to resolve day-to-day operational issues prior to the pandemic. In order to minimize the risk of viral transmission and COVID-19 disease within a transplant center’s workforce, efforts to reduce in-person meetings and conferences were rapidly adopted in the early months of the pandemic. In-person selection and listing conferences were transitioned to group virtual meetings (50). While there were initial technological challenges to surmount, digital platforms for clinical and administrative meetings became commonplace. In our center, one unintended benefit of this development was that pre-existing limitations in our transplant center physical space were overcome through the use of digital platforms. Everyone on the multidisciplinary transplant team could fit in one “room” when discussing patients, including nephrologists, surgeons, nurses, social workers, dieticians, pharmacists, other medical specialists, financial coordinators, living donor advocates, and administrators. Mirroring other industries, transplant centers could provide its staff with opportunities for remote work which provided much-needed flexibility to clinical and non-clinical personnel. Digital platform adoption for virtual meetings not only revolutionized patient interactions, but also interprofessional interactions as well.

## Perioperative care processes

### Preoperative infectious disease screening

Perhaps one of the most important early breakthroughs in the pandemic was the availability of antigen testing for transplant candidates, living donors, and deceased donors. Near-universal clinical testing in early 2020 was critical in assessing real-time safety to proceed with transplant, evaluating potential viral transmission through donation and transplant, and managing resources as test results influenced utilization of personal protective equipment (PPE), anesthesia and perioperative care processes, use of respiratory isolation protocols. Positive tests in transplant candidates at the time of organ offer are frequently associated with organ decline and transplant cancelation and reactivation on the waiting list after recovery from illness or 7–10 days if the transplant candidate is asymptomatic (51). In the post-transplant setting, testing impacts in-hospital and post-operative management (52–54).

Global practices varied in the early days of the pandemic. Deceased and living donor candidates with either COVID-19 exposure or positive tests were often excluded from donation, and some centers chose to rely more on local deceased organs where the regional COVID-19 rates were known and detection rate could be better trusted (30, 34). Many transplant hospitals applied triage systems to transplant candidates, opting in the early days of the pandemic and during disease surges to place living donor transplants and combined kidney-pancreas transplants on hold, as an example. While these decisions were largely driven by the availability of local hospital resources and clinical judgment of the perceived urgency of transplant, they are not without repercussions. In England, patients who are removed from the waitlist for clinical reasons that predate the onset of COVID-19 had worse patient and graft survival, even if they were transplanted within 5 years following suspension (4). More recent studies have indicated that practice changes related to COVID-19 has been associated with an increase in waitlist hospitalizations and deaths as well as deaths in those who were never able to be evaluated for transplant candidacy (55, 56).

### Response from the transplant community

Transplant professionals rapidly realized the need for consolidated best practice guidance in all aspects of clinical transplantation in the early pandemic. The American Society of Transplant Surgeons (ASTS) formed a COVID-19 Strike Force to compile evidence-based guidelines. As the United States entered isolation in March 2020, the Strike Force stated that the main goals at the time were (1) Protect healthcare providers, (2) Treat patients with the virus, and (3) Prevent transmission to others. Transplants were thus only performed in acute life-threatening situations, all living donations were suspended, and local procurements were recommended to prevent transmission of the virus to procurement teams during travel. As the pandemic has continued, and as we have gained knowledge about the virus, the Strike Force recommendations evolved. While procurement team safety was still paramount, COVID-19 no longer prohibited travel nor transplantation. Other recommendations respected autonomy of transplant teams in accepting donor organs with previous COVID-19 infection and urged focus on this factor as a consideration on overall organ quality. Recommendations considered donors non-contagious 10 days after the onset of symptoms and significant recovery of organ function was expected after the initial viral inflammatory response had abated. While living donation had been active in multiple centers after the first 3 months of the pandemic, it was endorsed by the Strike Force once again by June 2020 (57). With experience and time, societies and individual transplant centers became more comfortable with using organs from COVID-19 positive donors, both from donors with asymptomatic infection and death from other causes, as well as donors who died of COVID-19 disease. Protocols for the utilization of these organ donors were developed, but COVID-19 donor transmission in non-lung solid organ transplantation continues to be rare but is considered to be unknown (58, 59).

### Other guidelines from scientific experts and professional organizations and evidence

Multiple national organizations have set forth newer guidelines, however, all recommendations come with the caveat of having insufficient long-term evidence surrounding this novel virus (60).

The National Institute of Health (NIH), American Society of Transplantation and the Organ Procurement and Transplantation Network (OPTN) published similar guidelines with a few minor differences (61–63). PCR testing within 72 h of deceased organ procurement, ideally as close to organ recovery as possible, and testing within 72 h prior to surgery for living donors was recommended. The specimens should be from respiratory sites using PCR because rapid antigen and non-respiratory testing lack sufficient evidence. It is recommended to defer transplant if either the donor or recipient tests positive for COVID-19 or if infection is strongly suspected. According to the AST, those who have had resolution of symptoms but continue to test positive within 21–90 days of disease onset are considered low risk for transmission. Both the AST and OPTN state that a positive test 21–90 days after resolved COVID-19 symptoms, a positive test 10–21 days after symptom onset, asymptomatic donors with an incidental positive test, and resolved COVID-19 with a positive test > 90 days can all be considered for non-lung organ acceptance. Though the guidelines state organ acceptability from an infectious disease standpoint, a positive test 90 days after resolution of initial COVID-19 infection could potentially represent re-infection. Additionally, the AST states that death related to COVID-19 complications can also be considered for non-lung acceptance. Though an increasing number of non-lung organs from positive donors have not shown evidence of recipient transmission, with all cases, the risk of waitlist mortality should be weighed against mortality in donor-derived infection (Table 1). A negative rapid antigen test is unacceptable if there is suspicion for infection and a confirmatory PCR test is necessary in these cases.

**TABLE 1:** National organization recommendations for COVID-19 testing.

Organization	Donor recommendations	Recipient recommendations	Other statements
NIH	• All donors should be tested • Living donors: Monitor for symptoms and exposures in the 2 weeks before transplant. Test within 72 h of donation. • Deceased donors: Test within 72 h of death • COVID + donors should be deferred if possible	• All recipients should be tested • COVID + recipients should be deferred if possible	• COVID-19 transmission risk from donor to recipient is unknown
AST	• Deceased and living donors should be tested within 72 h of donation • Positive test 21–90 days after resolved COVID-19 are unlikely to transmit infection • Mild symptoms within 10–21 days after symptom onset with positive test are unlikely to transmit disease in non-lung transplantations • Asymptomatic donors with incidental positive testing and resolved COVID-19 with a positive test > 90 days can be considered for non-lung acceptance. • Death related to COVID-19 complications can be considered for non-lung acceptance		• PCR from a respiratory source is the best test • A positive test 90 days after resolution of COVID-19 symptoms can be re-infection
OPTN			• N95 or similar respirator use for health workers taking care COVID + or potentially COVID + patients • Rapid antigen testing is insufficient
BTS/NHS	• Vaccination is recommended for living donors but not mandatory	• In the absence of medical contraindications, patients on the waiting list should accept approved vaccine doses (including boosters)	

NIH, National Institutes of Health; AST, American Society of Transplantation; OPTN, Organ Procurement and Transplantation Network; BTS, British Transplantation Society; NHS, National Health Services.

### Vaccination policies: History, present, and solutions

#### History

When the coronavirus first emerged in Wuhan, China at the end of 2019, it was unexpectedly virulent and led to unprecedented lockdown measures in the country. Similar measures were taken when the virus arrived in the Western world, but with varying degrees of success in controlling the spread. While it became obvious that immunization would be necessary, vaccine development is also a daunting task, leading the US Department of Health and Human services to create “Operation Warp Speed” to expedite the process (64). Traditionally, vaccinations are inoculations of either deactivated or a live attenuated virus to stimulate human B lymphocytes to produce antibodies. However, time pressure and the novelty of the virus itself led to the development of messenger RNA that encode the blueprint of the SARS-CoV-2 spike protein. While the spike protein is foreign to us, it is abundant on the surface of the virus, allowing B cells in the vaccinated person to produce antibodies that can neutralize the virus (65–67). By mid-summer, the two leading companies, Moderna and Pfizer, published promising clinical trial results, showing that both vaccines induced anti-SARS-CoV2 immune responses in all participants (68, 69). A few months later, both vaccines received emergency use authorization (70).

Of course, immunology is much more complicated, and we present a simplified version of the evidence. In the aftermath of vaccination, hospitals and national studies continued to identify COVID-19 as the leading cause of death in solid organ transplant recipients after the onset of the pandemic, some within the first year of transplant but noted throughout the post-transplant course (71). In the solid organ transplant population, elevated age, use of antimetabolite medications, recent transplant, and receiving a kidney allograft are all indirectly proportional to the level of anti-spike antibody, an oft used surrogate for immunity after vaccination (72–74). The immune response of transplant patients was more muted compared to the general population. Poor post-vaccination humoral responses have led to France’s early adoption of a third vaccination injection in dialysis and in transplant patients (75). The NIH also has a clinical trial underway to study if immune responses to vaccination improve when immunosuppression medication is temporarily reduced in the days leading up to and following vaccination (76, 77). Though still unmatched to the general population, as boosters became available in the US, data shows that additional mRNA inoculations increase antibody titers for the majority of solid organ transplant patients (78, 79).

Despite our increasing molecular understanding of the virus, in the fall of 2021, a woman in Colorado with ESRD was denied kidney transplantation due to her and her living donor’s unvaccinated status, sparking national political debate and fierce media coverage (80–84). She was informed that her status on the waitlist would be inactivated if she were not to start the vaccination series within 30 days and vaccination refusal warranted removal from the kidney transplant list. She cited religious grounds and uncertainties about the vaccine itself as reasons for her hesitancy and reported feeling coerced by the hospital system. Despite the publicity, University of Colorado Health held firm on their decision, stating that patients undergoing organ transplant have higher mortality rates in unvaccinated organ recipients who contract COVID-19 and that transplant candidates are routinely subject to health requirements, including vaccinations as well as abstaining from tobacco and alcohol (85).

Though the media coverage of the Colorado case was intense, vaccine hesitancy long predates the pandemic. Vaccination discussions have historically centered around children. In recent years, parental refusal for non-medical reasons such as religion and uncertainties about the vaccine itself have become increasingly common in the general and transplant pediatric population (86–88). Some of this hesitancy can be linked to the now admittedly falsified studies linking vaccines to autism (89). The American Academy of Pediatrics and the American Society of Transplantation (AST) recommends complete appropriate vaccinations prior to solid organ transplantation, but up to 70% of children are incompletely vaccinated before surgery because most of the times, the children were either too young or too sick to be immunized at the appropriate ages (90, 91). When presented with a hypothetical situation of a parent refusing all vaccinations for non-medical reasons prior to solid organ transplant, programs responded very differently: 47% would list the patient, 22% would not, and 30% were unsure. Additionally, only 4% of pediatric transplant programs had a written policy regarding vaccine refusal (92).

#### Present

Most vaccination literature exists in the pediatric population, and adult practices lacked attention until the COVID-19 pandemic. While many transplant centers have developed a vaccination policy since the Colorado case, no strict national policy exists currently. The most recent joint statement with ASTS, AST, and the International Society of Heart and Lung Transplantation in March 2022, officially recommends all eligible transplant candidates, donors, and caretakers be vaccinated, ideally 2 weeks prior to surgery with two vaccinations plus boosters, but ultimately, individual centers should make the final decision and have clear and understandable policies (93). Proponents of having universal guidelines state that vaccinations protect the recipient and the allograft from vaccine-preventable disease, protects other patients and healthcare workers in the clinical setting, and maximizes the benefits of scarce organs. The opposition argue that vaccination requirements may cause irreversible harm to patients who refuse, violate patient autonomy, and create additional barriers to marginalized groups (94).

Similar discussions occurred in Europe. While persuasive arguments could be made for and against vaccine mandates, the NHS Blood and Transplant and the British Transplant Society urged physicians to discuss the benefits and repercussions of vaccine refusal, but ultimately do not mandate vaccination for transplant candidacy in a public joint statement in 2021 (95, 96).

Individual transplant programs have struggled with the impact of COVID-19-related mortality in their post-transplant patients (97). Since the pandemic, COVID-19 has been a leading cause of post-transplant mortality, particularly in patients with prolonged immunosuppression (98). Transplant outcome public reporting emphasize graft survival and patient survival in the first 3 years after transplant, and so many programs have developed COVID-19 vaccination policies to ensure continuation of strong post-transplant outcomes. Excess graft loss or death related to COVID-19 has significant impact on transplant programs with regard to quality rankings, which ultimately could affect their standing with commercial payers. The development of these policies has been met with controversy. COVID-19 vaccine mistrust has frequently been shaped as a political statement and vaccination rates have varied geographically. Transplant programs have received feedback from referring physicians and patients expressing their lack of trust or refusing vaccines altogether. Transplant programs have also had to contend with their own vaccine requirements—how to navigate the creation of a COVID-19 vaccine mandate for transplant candidacy if other vaccines (hepatitis B, MMR, etc.) were not absolutely required for listing. The pandemic has presented many challenges to clinical transplant program policy development and will likely do so as we evaluate epidemiologic trends in disease, clinical outcomes of COVID-19 with new variants, and whether low severity infections will become the norm as SARS-CoV-2 becomes endemic.

#### Solutions

In the US and globally, economically, and socially disadvantaged groups were disproportionally affected by COVID-19 infections (99, 100). Though African Americans represented 10% of the solid organ transplant population at one center, they made up 50% of the COVID-19 positive cohort (101). While vaccination requirements can potentially exacerbate preexisting mistrust in the healthcare system due to historical mistreatments of disadvantaged communities, interventions that attenuate the clinical impact of COVID-19 disease exist (102). For the extremely marginalized population, addressing their basic needs for survival such as access to food, shelter, and basic healthcare opens avenues for vaccination (103). Additionally, most early interventions for vaccination refusal have centered around the Information Deficit Model (104). Though correcting misinformation about vaccinations did not elicit behavioral change, a trusting relationship between patients, their caregivers, and providers that provides unbiased vaccine information increased vaccination rates in those diametrically opposed to immunization previously (105–108). In the modern era, social media has worked well to encourage the partially vaccinated to complete their series.

US Transplant programs have also developed clinical pathways to vaccinate patients for COVID-19 during the transplant evaluation phase through vaccination appointments with transplant center providers or piggybacking on other appointments to optimize patient convenience as well as obeisance to transplant center vaccination policies for candidates and living donors. Ultimately, transplant programs will need to closely monitor the progression of SARS-CoV-2 epidemiology to inform vaccine policy mandates. Furthermore, transplant programs are currently navigating the development of clinical pathways for utilization of monoclonal antibodies that serve as spike protein attachment inhibitors for the virus as pre-exposure prophylaxis (EVUSHELD™, tixagevimab co-packaged with cilgavimab) (109). The exorbitant mortality observed in 2020 and 2021 in the post-transplant population has given transplant programs the experience it needs to adapt with regard to policies and the availability of new therapeutics.

## Post-transplant care

### COVID-19 related post-transplant morbidity and mortality

From May 2021 to Feb 2022 in the US, 455 non-lung organs were transplanted from donors with a positive COVID-19 test, with 278 transplants being kidneys (62). So far, results of donor-derived COVID-19 transmission during transplantation are rare (110). In an Italian series, 10 urgent transplant need patients received livers from deceased brain death donors who either had positive COVID-19 testing at time of transplant or had previously been infected. Of the 10 recipients, 5 had history of severe COVID-19 infection requiring hospitalization before transplant. Eight tested negative and two tested positive for COVID-19 in the immediate preoperative setting using a respiratory source sample. The 8 patients who tested negative prior to surgery continued to test negative after transplantation with a potentially infected donor organ, and none of the 10 patients experienced any COVID-19 related symptoms (59). Another study showed that two recipients who were COVID-19 negative prior to transplant remained negative post-liver transplantation with organs from incidentally positive donors without known history of COVID-19 infection. Interestingly, one of the recipients was unvaccinated (111). Despite documented successes, it is unclear how protective vaccination (or even partial vaccination) in recipients is against organs from COVID-19 positive donors. What is clear is that herd immunity applies to small communities as well. Unvaccinated caretakers have an increased chance of spreading COVID-19 to recipients, and if infected, vaccinations lessen the severity of disease if the recipient contracts COVID-19 post-transplantation (58, 112).

Toward the beginning of the pandemic, studies in the United States cited mortality rates of 13–28% in kidney transplant recipients who subsequently test positive for COVID-19. Those who were hospitalized for COVID-19 related complications had even higher mortality rates (113). Even when patients survive, they still had an 8–21% chance of requiring renal replacement therapy, a rate much higher compared to those who were not infected (114). Dauntingly high mortality rates were also seen in European transplant centers, with mortality rates ranging from 5 to 36% (115, 116). Though most early studies were single-center experiences, the TANGO International Transplant Consortium compiled data from 12 centers across the United States, Spain, and Italy of kidney transplant recipients who tested positive for COVID-19 and reported 52% AKI, 29% respiratory failure requiring mechanical ventilation, and 32% mortality (99). The need for hemodialysis as well as graft loss are thought to be sequela of acute kidney injury (AKI), a frequent COVID-19 complication affecting up to 50% of all post-renal transplant patients (117).

Additionally, while transplant patients are at high risk for severe symptomatic COVID-19 infection due to chronic immunosuppression, their other medical comorbidities also contribute to their high mortality rates (118). Elevated age, diabetes mellitus, obesity, chronic heart disease, chronic kidney disease, lung disease, and longer dialysis were all associated with higher morality (99, 101, 119–123). While the exact immunological mechanisms are unknown, age and comorbidities, not immunosuppression, may be driving the high mortality rate (124). Despite a reduced humoral response in the setting of medically induced immunosuppression, the crude mortality rate for COVID-19 positive patients on hemodialysis (30%) was higher compared to those who underwent kidney transplantation (15%) as well as the general population (14%) in one study (125).

### Telemedicine in post-operative care

Clearly, post-transplantation patients still require careful monitoring and care. Fortunately, technology can help ensure post-transplant continuity of care by helping mitigate similar logistical challenges surrounding geography and finances that patients experienced before surgery (126, 127). Regular appointments are important for monitoring immunosuppression and progress post-operatively. Patients have been shown to have improved adherence and reduced readmission rates after transplantation when followed virtually. Additionally, this group had better disease-specific quality of life and quicker return to employment, which is potentially attributable to increased self-care responsibility (128). Interestingly, increases in self-accountability and self-management did not change patient perception of the quality of care received compared to traditional face-to-face appointments (129). Benefits can also extend to living donors. Nearly half of the transplant centers lose contact with > 75% of donors within 2 years post-donation due to costs to donors and out of date contact information, which has limited our understanding of long-term outcomes following living donation (130, 131). Information on safety metrics could change the way we care for living donors and potentially encourage more altruistic donations. Therefore, telemedicine presents exciting future opportunities and avoids donor financial burden related to travel in the present.

Technology also has limitations. Though most patients have access to smartphones and to the internet, individuals with minimal technology experience may require assistance in navigating the visit platform. So, this will necessitate transplant centers to have troubleshooting support available. There will also need to be legislation changes. Under the state of public health emergency, CMS as well as some private payors reimburse telemedicine visits across state lines at the same rate as an in person visit. However, access may change as the world moves away from the pandemic (132–134). Continued reimbursement of virtual/telemedicine care is a critical issue in solid organ transplantation as it has facilitated access to transplant as well as improved access to post-transplant and post-donation care. Virtual interfaces also limit parts of the clinical interaction, the ability to perform physical examination, and the possibility of weight-based medication dosing (135).

As the world readjusts to the new post-pandemic normal, the transplant community should continue to embrace the ability to deliver quality care through telemedicine. While the strain on hospital access and social distancing and isolation may lessen, patients in remote areas of the country will still require access to transplant centers. These can be opportunities to explore and to develop acute hospital at home models, which have been shown to reduce readmissions and cost while improving patient experience following non-transplant surgeries (136–139).

### Current practice recommendations

At our high-volume transplant center, donors and recipients are tested according to national society guidelines prior to transplant, which continue to change. For living donors and all recipients, vaccination is highly encouraged and an important part of the preoperative candidacy evaluation. Our institutional practice has also changed overtime. Rather than performing routine biopsies from all COVID-19 positive donors, procurement biopsies are now being used in a case-by-case basis to add reassurance in utilizing organs from COVID + donors that have significant clinical symptoms. Post-operative biopsies are typically reserved for evidence of organ dysfunction or injury. Recipients are monitored daily for symptoms related to COVID-19 for the next 14 days but are dismissed from the hospital if clinically able. If the patients are discharged before 2 weeks, then they are encouraged to isolate and to self-monitor for symptoms. Blood samples are drawn at regular intervals (2, 7, 14, 21, and 28 days) for detection of spike protein and nucleocapsid antibodies. Routine PCR antigen tests are also performed on respiratory samples. Patients receive monoclonal antibody post-exposure prophylaxis as well as empiric antiviral treatments.

When outpatient, our institutional practice is to reduce immunosuppression for 2–4 weeks when patients who test positive for COVID-19. Those who are admitted for symptomatic infections will have discontinuation of their antiproliferative medication, trough level measurements for tacrolimus/cyclosporine, daily steroid use, and infectious disease consultation. In general, off label use of hydroxychloroquine for prophylaxis was specifically not recommended due to lack of data, shortages, and risk of QT prolongation.

At the peak of the pandemic, many hospitals, including ours, adopted a no visitation policy to prevent community transmission (140). As healthcare organizations reopened from the pandemic, our hospitals allowed limited visitors (1–2 per day) for COVID-19 patients. Visitors would be screened for symptoms prior to entering the hospital, asked about exposures, and have their temperature taken. While visiting, they are required to wear a mask and be limited to the confines of the patient’s room (141). Patients who test positive for the virus would not be able to have in-person visitors.

In conclusion, the global pandemic has been challenging for kidney transplant programs worldwide. Policy changes in the United States have helped mitigate some of the cost and access issues to healthcare. Research has prevented deaths in the form of rapid vaccine development and helped in the management of COVID-19 related complications in the transplant population. Despite the hardships, the pandemic also brought forth practice changes that can help with longstanding problems of access through more widespread use of telemedicine. As the world emerges from the shock of the pandemic, we must carry forward the lessons we have learned and the tools we have garnered.

## Author contributions

CZ and AM: conception, design, and draft manuscript preparation. CZ: literature review. All authors reviewed and approved the final version of the manuscript.
